# Overexpression of chaperonin containing T-complex polypeptide subunit zeta 2 (CCT6b) suppresses the functions of active fibroblasts in a rat model of joint contracture

**DOI:** 10.1186/s13018-019-1161-6

**Published:** 2019-05-09

**Authors:** Xiaoyou Yi, Zhe Wang, Jianhua Ren, Ze Zhuang, Kaihua Liu, Kun Wang, Ronghan He

**Affiliations:** Department of Orthopaedic Surgery, The Third Affiliated Hospital of Sun Yat-sen University, No.600 Tianhe Road, Tianhe District, Guangzhou, 510000 China

**Keywords:** Chaperonin, CCT6b, CCT7, Anti-fibrosis, Joint contracture

## Abstract

**Background:**

Joint contracture is a fibrous disease characterized as joint capsule fibrosis that results in joint dysfunction and disability. The purpose of this study was to analyze the biological activities of chaperonin containing T-complex polypeptide (CCT) subunits and to determine the role of CCT chaperone in joint contracture in a rat model.

**Methods:**

In this study, the rat model of joint contracture was established by immobilizing the rat knee for 8 weeks. Then, fibroblasts were isolated from the posterior joint capsule and were cultured for functional analysis such as qRT-PCR, Western blot, transwell assay, and collagen assay. The effect of CCT subunit was determined by employing a lentivirus containing target gene and transfecting it into fibroblasts.

**Results:**

Results of qRT-PCR and Western blot showed that among all CCT subunits, CCT6b significantly decreased in the fibroblasts from contractive joints compared to cells from normal joints (*p* < 0.05). Overexpression of CCT6b by transfection of lentivirus containing CCT6b gene to active fibroblasts significantly inhibited fibrous marker (α-SMA, COL-1) expressions, fibroblast migration, and collagen synthesis (all *p* < 0.05). Moreover, fibrosis-related chaperone CCT7 expression was decreased with CCT6b overexpression (*p* < 0.05).

**Conclusion:**

The biological activities of CCT subunits in fibroblasts from the joint contracture rat model were analyzed in this study. CCT6b significantly decreased in the active fibroblasts, and overexpression of CCT6b significantly inhibited fibroblast functions. These findings indicate that CCT6b appears to be a potential molecular biomarker and therapeutic target for the novel therapies of joint contracture.

**Electronic supplementary material:**

The online version of this article (10.1186/s13018-019-1161-6) contains supplementary material, which is available to authorized users.

## Introduction

Joint contracture, characterized as losing active and passive range of motion (ROM) on the affected joints, is a severe complication resulting in irreversible disability after joint surgery or longtime immobilization [1]. Patients who suffered with joint contracture are restricted of joint movement and daily living, leading to permanently handicapped, which burdens family care and medical expenses [2]. The traditional treatment of joint contracture is through physiotherapy and stretching the affected joint; however, only 36% of the patients can achieve full recovery of ROM [3–5]. Novel therapies for addressing joint contracture emerge as an urgent need to recuperate joint mobility. Unfortunately, as the exact biological mechanism of joint contracture remains unclear, an effective therapy is yet to be proposed. The possible pathogenies of joint contracture include the deposition of extracellular matrixes in joint capsule, active fibroblast proliferation, and α-smooth muscle actin (α-SMA) expression [6]. Genetic lineage tracing technology indicated that during fibrous development, the resident active fibroblasts around the joint may be the main contributable cells [7]. Molecules that target active fibroblast functions are possible options to intervene the progression of joint contracture at the cellular level.

At the cellular level, we have previously confirmed that chaperonin containing T-complex polypeptide (CCT) subunit eta (CCT7) was a potential marker in joint contracture in the rat model [8]. The CCT chaperonin family, consists of eight subunit complexes in two stacked rings arrange as CCT 1-4-2-5-7-8-6-3-(1), functions as assisting folding and refolding newly synthesized proteins [9]. Most researches of CCT mainly focus on oncology and molecular biology, and each subunit shows a different contribution to cell motility, proliferation, apoptosis, and genome instability [10–12]. For example, CCT5 and CCT6 both play essential roles in actin and tubulin folding. CCT6 is found to have pathophysiological relevance in fibrous disease [13, 14]. Previous research showed that CCT6 was significantly increased in mucosal wounds, which may affect the fibroblast functions [15]. Moreover, CCT6 contains two subunits: 6a and 6b [16]. Inhibition of CCT6a can efficiently inhibit transforming growth factor-β (TGF-β, a fibroblast activator) signaling pathway [17]. The eight subunits may contribute to the proliferation and activation of fibroblasts since they cooperate in many biological functions [18]. Nevertheless, the biological functions of CCT subunits have not yet been systematically analyzed in joint contracture.

The goal of this study is to investigate the expressions and functions of eight subunits of CCT chaperonins in fibroblasts in a rat model with knee joint contracture. The hypothesis of our study is that changes of CCT chaperonins exist in the progress of joint contracture, and the alteration of CCT chaperonins can suppress the functions of active fibroblasts in a rat model of joint contracture.

## Materials

**Fig. 1:**
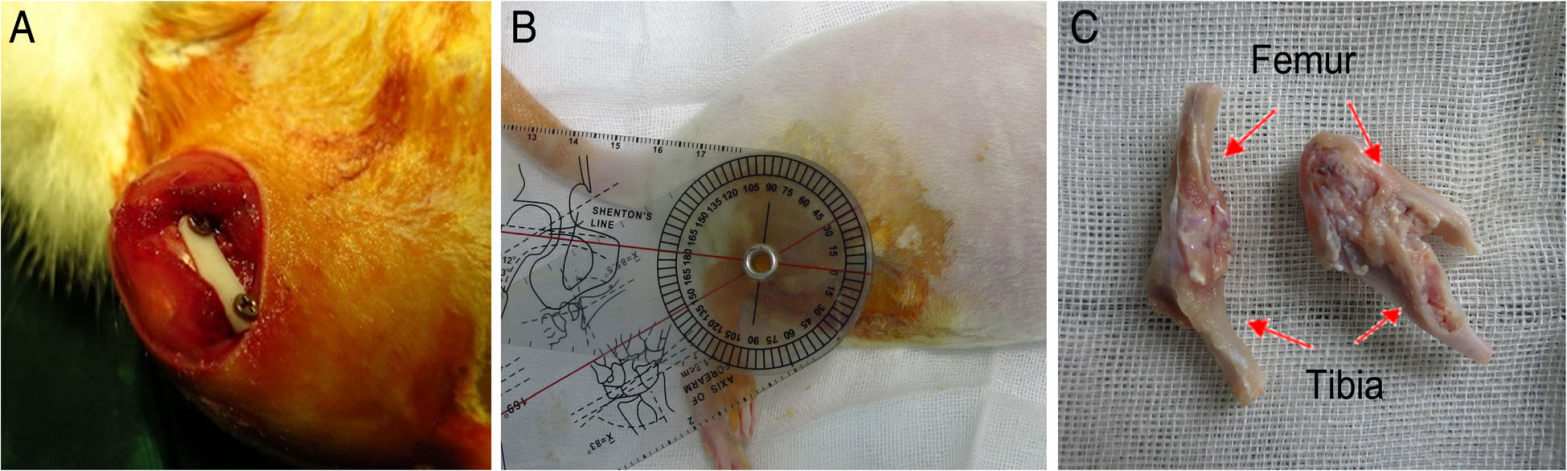
The immobilization-induced joint contracture rat model was established. **a** Rat right hind limb was immobilized by internal fixation to induce joint contracture. **b** After 8 weeks of immobilization, total knee extension range of motion (ROM) was analyzed. **c** Joint contracture was observed in the rat right limb after the skin, muscle, and plastic plate of the femur and tibia were removed

This animal study was conducted with the permission of the Institutional Animal Research Committee Approval of the Third Affiliated Hospital of Sun Yat-sen University. Twelve Sprague-Dawley (SD) male rats aged 16 weeks, weighing 280 ± 20 g, were purchased from the Animal Center for Medical Experiment of Guangdong. Joint contracture model procedures were performed as our previous study [8]. In brief, after 50 mg/kg sodium pentobarbital anesthesia, the right knee joints were immobilized by a polyformaldehyde plate (20 mm × 4 mm × 2 mm) and two metal screws (1.2 mm × 16 mm) at 45° flexion for 8 weeks (Fig. 1a, b). The fixed right knees were immobilized rigorously while the non-operative left knee joints could move freely. The left knees were taken as the control group. The rats were administered with sodium salicylate (150 mg/kg) for pain control. Eight weeks after immobilized surgery, rats were executed. The ROMs of joints were analyzed by an arthrometer as described by our group [8]. The torque value of the arthrometer was set at angles reached at 667 g/cm. The skin, muscles, and plastic plates were removed while post-capsules of the knee joint were carefully harvested (Fig. 1c).

Rat posterior capsule tissues were harvested and transferred to a super-clean bench immediately. Tissues were washed with phosphate-buffered solution (PBS; KeyGen, China) thoroughly. Then, tissues were sub-sectioned to 1 mm × 1 mm and placed in culture flasks. After 4-h incubation at 37 °C, 5 mL of the growth medium of Dulbecco’s modified Eagle’s medium (DMEM; KeyGen) supplemented with 10% fetal bovine serum (FBS; PAN) was added into the flasks to culture fibroblasts. Fibroblasts were then placed in a 37 °C incubator supplied with 5% CO_2_ and 95% humidity. The medium was changed every couple of days. Fibroblasts were then divided into three groups: fibroblasts derived from the contralateral knees (CF), the immobilized knees (IF), and IF cells treated with lentivirus (IF-L).

Lentivirus containing CCT6b gene was constructed by KeyGen Corporation according to the manufacturer’s protocol. Gene sequence of CCT6b was found in the NCBI gene database (ID: 12467). Primers were designed and synthesized according to the gene sequence. CCT-6b gene fragment was amplified by qRT-PCR, and the restriction enzyme locus of the gene fragment was analyzed. Then, CCT6b gene fragment was linked to lentivirus vehicle after digestion of restriction enzyme. The production was added to competent cell, and then the monoclonal colony was verified by qRT-PCR to select lentivirus vehicle. Lentivirus vehicle and packaging plasmid were extracted and transferred. The supernatant liquid of transferred fibroblasts was collected and concentrated after 48-h cell culture to obtain enriching lentivirus liquid. Fibroblasts from the IF group were washed with PBS, then diluted lentivirus solution was added and incubated at 37 °C with 5% CO_2_ for 8 h. After incubation, the cell medium was changed with a new diluted lentivirus solution and incubated for 24 h. Then, the fibroblasts were switched to the original growth medium for 48 h. After transfection, total protein was extracted from the fibroblasts, and Western blot assays were employed for verifying CCT6b expression.

The relative gene expressions of α-SMA, COL-1, and CCT subunits were characterized by qRT-PCR in fibroblasts from CF, IF, and IF-L groups. The qRT-PCR was manipulated as described in the previous work [8]. In brief, when fibroblasts reached 90% confluence, total mRNA extractions were conducted by Trizol reagent (Ambion, USA) according to the manufacturer’s instructions. The total mRNA concentration was determined by a NanoDrop1000 spectrophotometer (Thermo Scientific, USA). qRT-PCR was then performed utilizing One Step SYBR PrimeScript RT-PCR Kit (Takara, Japan) on ABI 7500Fast PCR system (Applied Biosystems, USA). The relative expressions of the target genes were normalized to the reference gene GAPDH. The sequences of the primers used are summarized in Additional file 1: Table S1.

Total proteins of fibroblasts from CF, IF, and IF-L groups were obtained by M-PER mammalian protein extraction reagent (Thermo Scientific, USA). The concentrations of the total proteins were detected by the BCA Protein Assay kit (Thermo Scientific, USA). Protein from the three groups was resolved by SDS-PAGE loading buffer according to its concentration and analyzed by immunoblotting with specific antibodies for CCT1, CCT2, CCT3, CCT4, CCT5, CCT6a, CCT6b, CCT7, CCT8, α-SMA, and COL-1 (ABclonal, USA). GAPDH was used as an internal reference. The signals were analyzed by SuperSignal West Femto Trial Kit Prod (Thermo Scientific, USA).

Fibroblasts from CF, IF, and IF-L groups were seeded in six-well plates (6 × 10^5^ cells/well) cultured in a growth medium supplemented with 50 mM of ascorbic acid (Wako Pure Chemical Industries, Japan) for 10 days. After the removal of the medium, the extracellular collagen produced by the fibroblasts was extracted by incubating each substrate in 1 mL of pepsin solution (0.1 mg/mL in 0.5 M acetic acid) overnight at 4 °C. The extracted collagen was quantified utilizing a Sircol™ Collagen Assay kit (Biocolor, UK) as per the manufacturer’s instructions.

Fibroblasts from CF, IF, and IF-L groups were investigated by transwell assay to evaluate their migratory abilities. Fibroblasts were starved in DMEM media without FBS for 12 h. Then, cells were digested by 0.25% trypsin-EDTA solution and washed by DMEM media again. 5 × 10^4^ of fibroblasts were seeded in the upper chamber while the lower chamber was added DMEM media with 10% FBS as an attractant. After 24-h incubation in 37 °C with 5% CO_2_, the chamber was washed with PBS and then fixed with paraformaldehyde. 0.1% crystal violet staining solution (KeyGen, China) was utilized for cell staining. The number of fibroblasts that migrated across the membrane was counted at × 100 magnification for five microscopic fields.

At least three samples for each experimental condition were investigated. A one-way analysis of variance (ANOVA) was utilized to analyze the data. Significant difference was accepted at *p* < 0.05.

## Results

**Fig. 2:**
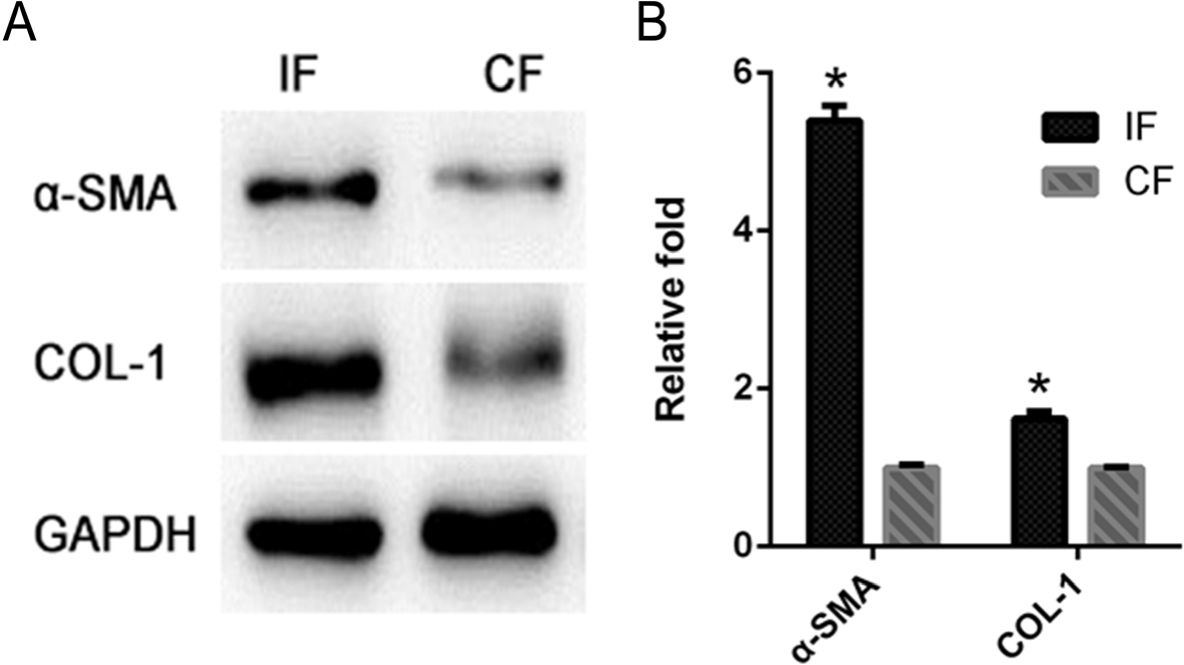
Protein and mRNA expressions of fibrous markers (α-SMA, COL-1) in fibroblasts from the IF and CF groups. **a** Western blot using specific antibodies for α-SMA and COL-1. **b** qRT-PCR results of α-SMA and COL-1 were shown. The asterisk indicates *p* < 0.05 compared to the CF group. IF fibroblasts derived from the immobilized knees, CF fibroblasts derived from the contralateral knees. GAPDH was used as the internal reference in the Western blot

Twelve rats were included in joint ROM measurement. None of the rats was found to be dead and infected or to have immobilization failure. As shown in Table 1, the angles missing to reach complete extension (0°) of the contracture knees (82.3 ± 6.5°) were significantly bigger than the non-immobilized control group (20.7 ± 8.2°) (*p* < 0.05). Results of qRT-PCR and Western blot indicated that both mRNA and protein levels of fibrous markers (α-SMA, COL-1), in fibroblasts of the IF group, were significantly increased (*p* < 0.05) when compared to those of the CF group (Fig. 2). Therefore, the joint contracture model was successfully established.

**Table 1:** Range of motion in the extension of rat knees

Groups	Number of joints	Mean lack of knee extension ± SD (°)
Immobilized	12	82.3 ± 6.5*
Contralateral	12	20.7 ± 8.2

Range of motion measurement corresponds to the angle missing to reach complete extension (0°) at the knee; torque = 667 g/cm

*SD* standard deviation

*Significant difference (*p* < 0.05) compared with the contralateral knee

**Fig. 3:**
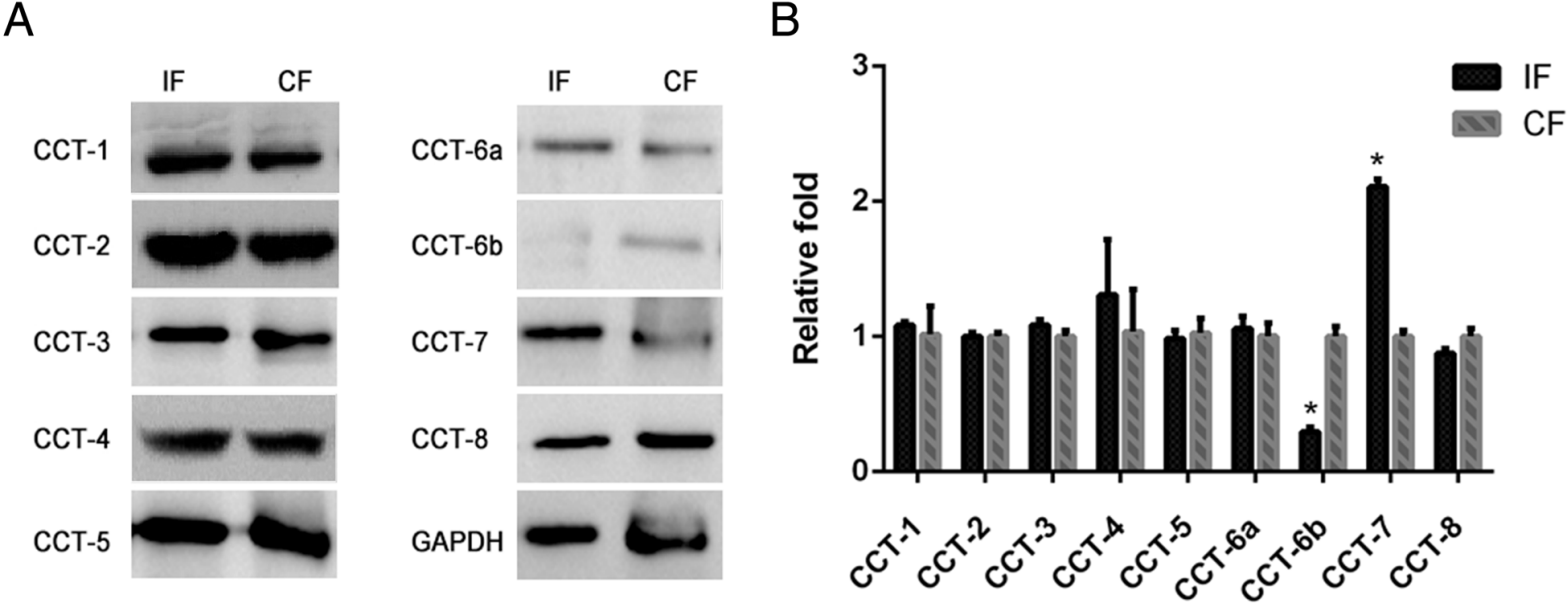
Protein and mRNA expressions of all subunits of the CCT family in fibroblasts from the IF and CF groups. **a** Protein expressions of CCT1, CCT2, CCT3, CCT4, CCT5, CCT6a, CCT6b, CCT7, and CCT8. **b** qRT-PCR results of all subunits of the CCT family. The asterisk indicates *p* < 0.05 compared to the CF group. IF fibroblasts derived from the immobilized knees, CF fibroblasts derived from the contralateral knees. GAPDH was used as the internal reference in the Western blot

We examined both the protein and mRNA levels of CCT subunits in fibroblasts of the IF and CF groups by Western blot and qRT-PCR. Results of the Western blot indicated that among all CCT subunits, the expressions of CCT6b and CCT7 were significantly different in the IF group compared to the CF group (*p* < 0.05), while other CCT subunits showed no difference between the two groups (Fig. 3a). Comparing to the CF group, CCT6b expression was significantly decreased in fibroblasts from the IF group, while an increased expression of CCT7 was observed (*p* < 0.05). qRT-PCR also demonstrated the similar trends of CCT6b and CCT7 mRNA expressions (*p* < 0.05) in the IF group when compared to the CF group (Fig. 3b).

**Fig. 4:**
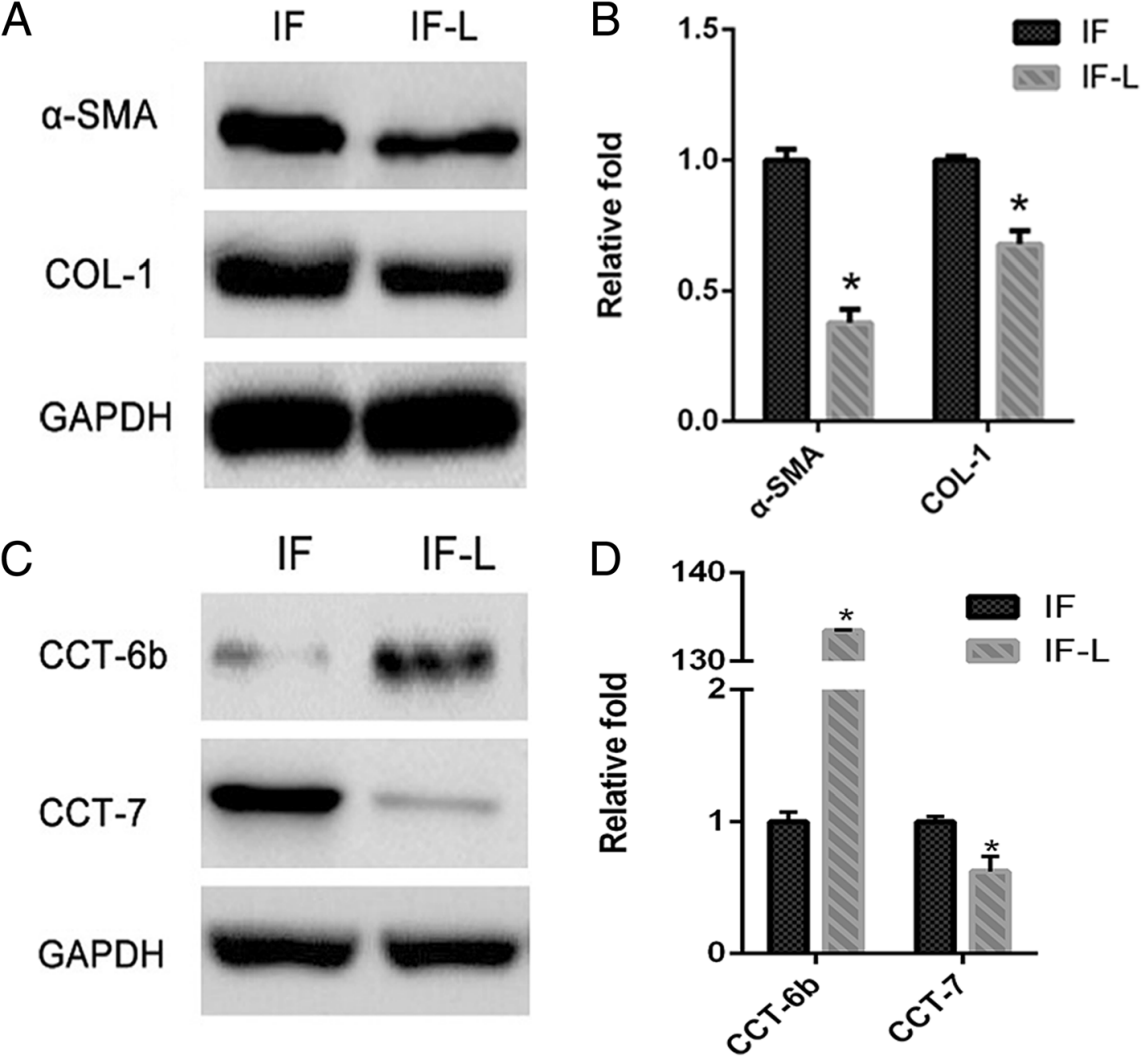
Transfection of lentivirus containing CCT6b gene to active fibroblasts significantly inhibited fibrous marker expressions. **a** Protein expressions of α-SMA and COL-1 in the IF-L group and IF group. **b** Relative mRNA expressions of α-SMA and COL-1 in the IF-L group compared to the IF group. The asterisk indicates *p* < 0.05 compared to the CF group. **c** Western blot results of CCT6b and CCT7 subunits in the IF and IF-L groups. **d** Relative quantitation of mRNA of CCT6b and CCT7 in the IF and IF-L groups was shown. IF fibroblasts derived from the immobilized knees, CF fibroblasts cells derived from the contralateral knees, IF-L fibroblasts derived from cells treated with overexpressed CCT6b lentivirus. GAPDH was used as the internal reference in the Western blot

We employed lentivirus overexpressed CCT6b gene to upregulate the CCT6b mRNA and protein expression levels in fibroblasts of the IF group. CCT6b protein and mRNA levels in the IF-L group were increased (*p* < 0.05, Fig. 4c, d) compared to those in the IF group. These results indicated that the lentivirus overexpressed CCT6b effectively increased the expression of CCT6b. Analysis of mRNA and protein expressions of α-SMA and COL-1 in fibroblasts from the IF-L group revealed a significant reduction (*p* < 0.05) compared to fibroblasts from the IF group (Fig. 4a, b).

**Fig. 5:**
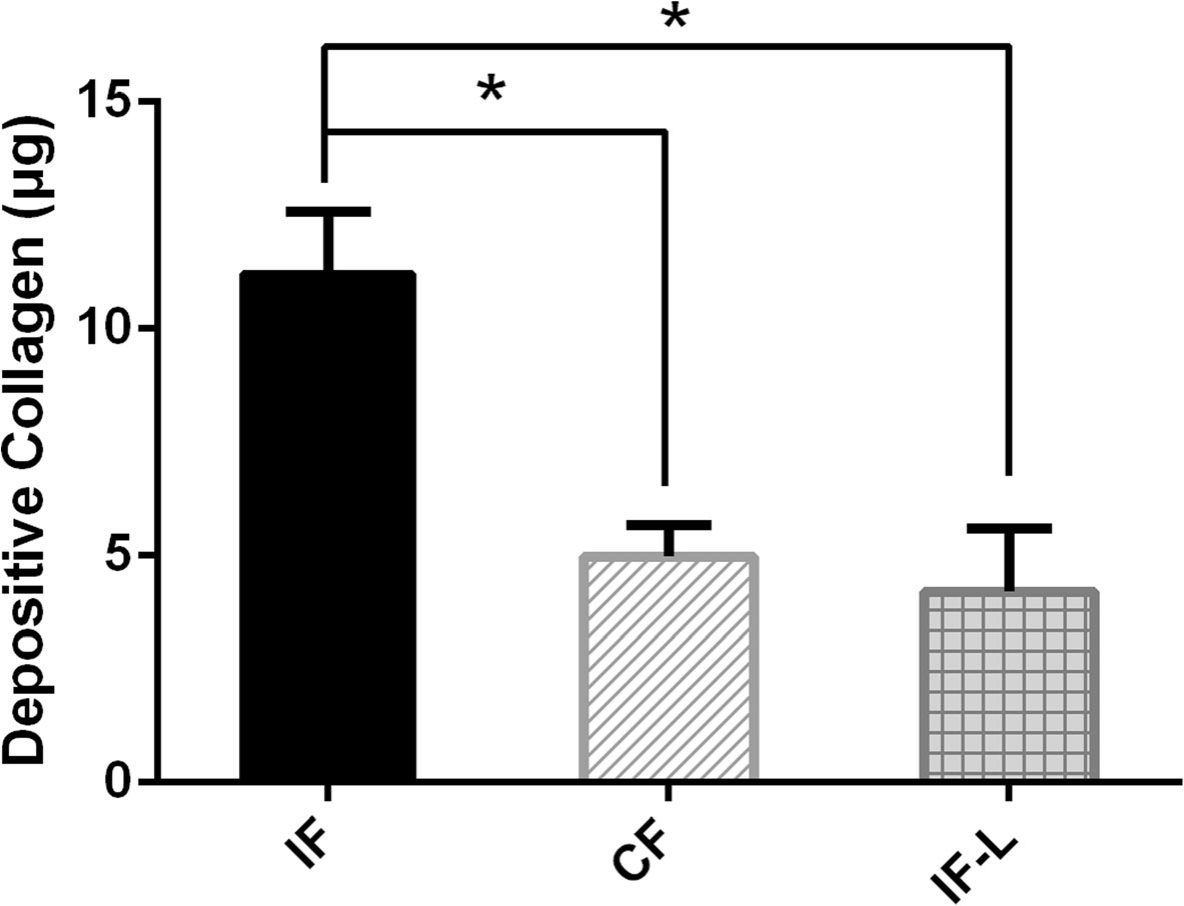
The effect of CCT6b overexpression on fibroblast collagen synthesis in active fibroblasts from the IF and IF-L groups. The asterisk indicates *p* < 0.05 compared to the IF group. IF fibroblasts derived from the immobilized knees, CF fibroblasts cells derived from the contralateral knees, IF-L fibroblasts derived from cells treated with overexpressed CCT6b lentivirus

**Fig. 6:**
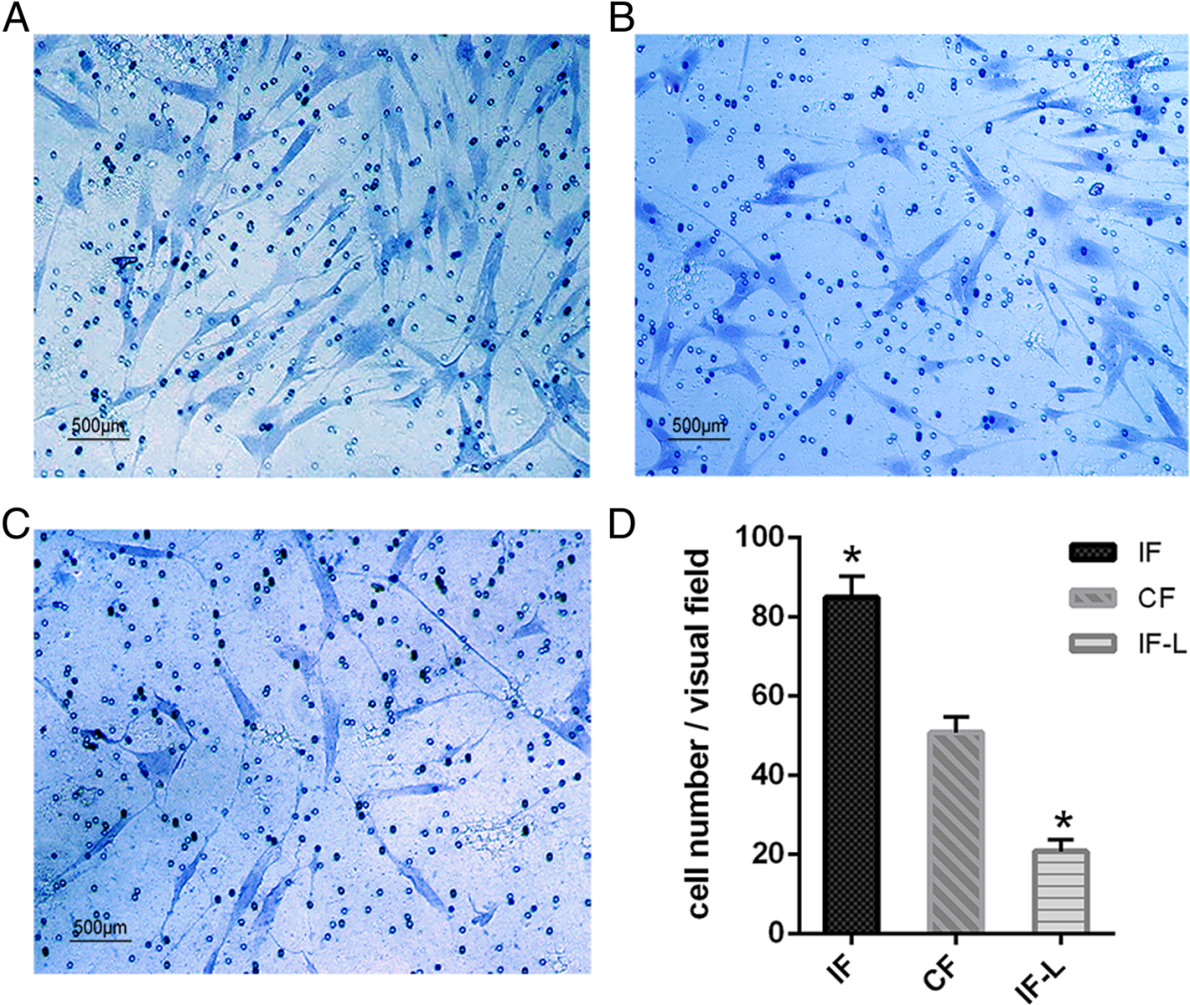
Transwell assay of fibroblasts derived from the IF group (**a**), CF group (**b**), and IF-L group (**c**). Scale bar = 500 μm. **d** Quantification of the migrated cells per microscopic field at × 100 magnification (*n* = 5). The asterisk indicates *p* < 0.05 compared to the CF group. IF fibroblasts derived from the immobilized knees, CF fibroblasts cells derived from the contralateral knees, IF-L fibroblasts derived from cells treated with overexpressed CCT6b lentivirus

To investigate the effect of CCT6b overexpression, we checked the fibroblast collagen synthesis and migration. In the collagen assay, fibroblast collagen synthesis in the IF-L group was significantly decreased when compared to that in the IF group (*p* < 0.05, Fig. 5). Transwell assay indicated that active fibroblasts from the IF group showed higher (*p* < 0.05) migratory ability compared to those from the CF group (Fig. 6). Importantly, overexpression of CCT6b by lentivirus in the IF-L group can significantly suppress fibroblast migration compared to that in the IF group (Fig. 6) (*p* < 0.05).

We analyzed the protein and mRNA expressions of CCT7 subunit in the IF and IF-L groups. Western blot result indicated that the expression of CCT7 was significantly diseased (*p* < 0.05) in the IF-L group compared to the IF group, when CCT6b was overexpressed (Fig. 4c). qRT-PCR also shown similar changes of CCT6b and CCT7 mRNA expressions (Fig. 4d).

## Discussion

Our results indicated that CCT6b played an anti-fibrous role in the regulation of active fibroblasts in a rat model of joint contracture. Joint contracture is a severe complication after external joint immobilization. However, the main mechanisms of the fibrous joint capsule are still unknown. The effect of traditional treatment of joint contracture is unsatisfied. Thus, novel therapies emerge as an urgent need to orthopedic surgeons and researchers. The active fibroblasts, acting as the main pathogenic cells for joint contracture, can be the crucial cellular target for anti-fibrous treatment [19, 20]. Fibroblasts are activated by fibrous factors, such as TGF-β, and express fibrous markers α-SMA and COL-1 [6]. Enhanced deposition of extracellular matrix, particularly type I collagen molecule, is essential in the progress of joint contracture [21]. The balance of extracellular matrix generation is interrupted in joint contracture [22]. Post joint capsule is considered as the predominant tissue of joint contracture [7]. As shown in our rat model, the ROM data indicate that the joint contracture rat model has been successfully established. We explored this study, in the case of CCT subunits described here, by analyzing each CCT subunit to establish the extent of the individual function of CCT subunits in joint contracture.

CCT is a group II chaperonin complex consisted of two back-to-back hetero-octameric rings which assist the folding of actin or tubulin substrates [23]. Different CCT subunits are arranged in a confirmed order in each ring, and researches indicated that each subunit of CCT had distinct substrate specialization [23]. It has been reported that α-actin, an important actin, folded with the assistance and interaction of CCT2, CCT4, and CCT5 [23]. CCT2 and CCT5 also have been shown to be the primary functional subunits for tubulin that interacted with CCT7 and CCT8 [23]. In our previous study, CCT7 was reported to be overexpressed in fibroblasts from the joint contracture rat model and the suppression of CCT7 can significantly induce anti-fibrous effect [8]. In this study, all CCT subunits were analyzed in a joint contracture rat model to observe the function of each CCT subunit during the pathological process of joint contracture. Both Western blot and qRT-PCR results indicated that CCT6b may be a chaperonin subunit which functions as anti-fibrous CCT subunit.

CCT6b was a subunit of CCT6, and CCT6b has been found to be significantly increased in mucosal wounds, which may affect the fibroblast functions [24]. A recent study by Ying et al. showed that CCT6a, another subunit of CCT6, can suppress SMAD2 and promote prometastatic TGF-β signaling pathway in carcinoma [17]. According to our knowledge, few researches have focused on the specific function of CCT6b in fibrous disease. In our experiments, fibroblasts from the IF group presented lower CCT6b expression compared to those from the CF group. To further investigate the function of CCT6b in joint contracture, overexpression of CCT6b simultaneously suppressed α-SMA and COL-1 in fibroblasts. Thus, CCT6b appears to be a novel and potential molecular biomarker and therapeutic target of joint contracture

The fibroblast migration and collagen synthesis were indispensable in fibrous disease such as joint contracture [24]. During the process of joint contracture, fibroblasts were observed to migrate and accumulate around the joint capsule in sever joint contracture patients [25]. The results of our transwell assay demonstrated that CCT6b could function as an inhibitor of cell migratory ability. During the joint contracture procedure, the collagen synthesis of fibroblasts was considered as an essential factor [7]. These cell collagen assay data indicated that CCT6b not only could inhibit the expression of fibrous markers α-SMA and COL-1, but also could suppress the collagen synthesis of active fibroblasts.

Although the CCT6b can suppress the functions of active fibroblasts, the anti-fibrous mechanism of CCT6b was still unclear. As CCT7 has been confirmed to function as a fibrosis-related chaperone in joint contracture [8], we explored CCT7 subunits in the IF-L group to further analyze the effects of CCT6b overexpression. Interestingly, in the IF-L group, fibrous marker CCT7 was inhibited with the overexpression of CCT6b. This result suggested that CCT6b and CCT7 play contrary roles in the pathology of joint contracture, and the balance of CCT6b and CCT7 may be a crucial factor in joint contracture. However, further investigation is still needed to further explain this phenomenon.

## Conclusion

The biological activities of CCT subunits in fibroblasts from the joint contracture rat model were analyzed in this study. CCT6b significantly decreased in the active fibroblasts, and overexpression of CCT6b significantly inhibited fibroblast functions. These findings indicate that CCT6b appears to be a potential molecular biomarker and therapeutic target for the novel therapies of joint contracture.

## Additional file

Additional file 1: **Table S1.** List of siRNA sequences used in this study. **Figure S1.** Immunohistochemistry analysis of the expression of CCT6b in human contractive knee capsule (A) and normal control sample (B). Arrows indicate the CCT6b staining (magnification × 10). (DOCX 2594 kb)

## Abbreviations

CCT: Chaperonin containing T-complex polypeptide
CF: Fibroblasts derived from the contralateral knees
COL-1: Collagen-1
DMEM: Dulbecco’s modified Eagle’s medium
FBS: Fetal bovine serum
IF: Fibroblasts derived from the immobilized knees
IF-L: IF cells treated with lentivirus
PBS: Phosphate-buffered solution
qRT-PCR: Quantitative real-time polymerase chain reaction
ROM: Range of motion
TGF-β: Transforming growth factor-β
α-SMA: α-Smooth muscle actin
