# Supplementary material for: Overexpression of chaperonin containing T-complex polypeptide subunit zeta 2 (CCT6b) suppresses the functions of active fibroblasts in a rat model of joint contracture
Source: J Orthop Surg Res. 2019 May 9;14:125. doi: 10.1186/s13018-019-1161-6 (PMC6507144; doi:10.1186/s13018-019-1161-6)
Supplement: Supplementary file 1 — Table S1. List of siRNA sequences used in this study. Figure S1. Immunohistochemistry analysis of the expression of CCT6b in human contractive knee capsule (A) and normal control sample (B). Arrows indicate the CCT6b staining (magnification × 10). (DOCX 2594 kb) [file 13018_2019_1161_MOESM1_ESM.docx]

**Supplementary Content**

**Overexpression of chaperonin containing T-complex polypeptide subunit zeta 2 (CCT6b) suppresses the functions of active fibroblasts in a rat model of joint contracture**

**Xiaoyou Yi† · Zhe Wang† · Jianhua Ren · Ze Zhuang · Kaihua Liu · Kun Wang* · Ronghan He***

**Department of Orthopedic Surgery, the Third Affiliated Hospital of Sun Yat-sen University, 510000, Guangzhou, China**

† These authors contributed equally to this work.

* Correspondence should be addressed to Kun Wang (email: wangk@mail.sysu.edu.cn) or Ronghan He (email: [herh3@mail.sysu.edu.cn](mailto:herh3@mail.sysu.edu.cn))

Other authors’ emails: Xiaoyou Yi (1301428198@qq.com), Zhe Wang (150248783@qq.com), Jianhua Ren (renjianhua001@126.com), Ze Zhuang (zzbjm@163.com), and Kaihua Liu (liukh3@mail.sysu.edu.cn).

No.600 Tianhe Road, Tianhe District, 510000, Guangzhou, China

Tel: +86 020-85252229

# **Table S1** List of siRNA sequences used in this study.

| Gene | Sequence (5'-3') |
| --- | --- |
| a-SMA | F: GTCCCAGACATCAGGGAGTAA |
|  | R: TCGGATACTTCAGCGTCAGGA |
| COL-1 | F: GCTCCTCTTAGGGGCCACT |
|  | R: CCACGTCTCACCATTGGGG |
| CCT1 | F: AAGCGACAATGTTGGGACAAG |
|  | R: TCCAAAACTCTCTTCACCACAC |
| CCT2 | F: CTTTCCCTCGCACCTGTTAAT |
|  | R: GGCACCGATAAACGACGACA |
| CCT3 | F: GGACCTGCTTGGGACCTAAAT |
|  | R: CGGGATGCTGGACTTGAATC |
| CCT4 | F: ACCACGTCGGTTGTCATCATT |
|  | R: CAGTTGCACAGGTCGAGACAT |
| CCT5 | F: ACCCTCGCCTTCGATGAGTAT |
|  | R: GCATTGTGTTTGCTACAGCTTT |
| CCT6a | F: GCGGCGGTAAAGACCCTAAAT |
|  | R: CCCAAGTTGGTCCTCAGAACAT |
| CCT6b | F: CAACACCCAACGGCTTCCAT |
|  | R: CAGGACATTTGAGGTAGTGCC |
| CCT7 | F: CCAGTTATCCTGTTGAAAGAGGG |
|  | R: GACCCAGGGTGGTTCTTACA |
| CCT8 | F: ATTTCTCGGGATTAGAAGAGGCT |
|  | R: CCAGGCGATTGATGACCATTT |
| GAPDH | F: AGGTCGGTGTGAACGGATTTG |
|  | R: TGTAGACCATGTAGTTGAGGTCA |


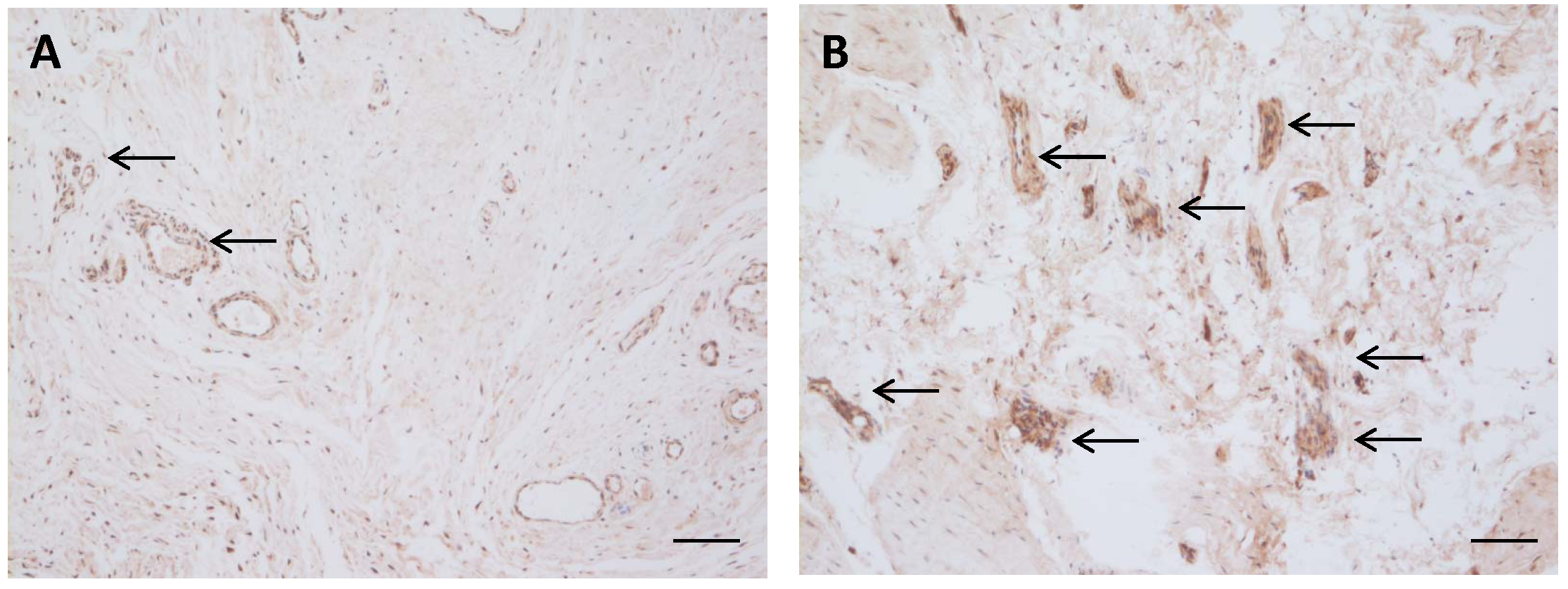


**Figure S1.** Immunohistochemistry analysis of the expression of CCT6b in human contractive knee capsule (A) and normal control sample (B). Arrows indicate the CCT6b staining (magnification: 10×).
